# Novel GFP-fused protein probes for detecting phosphatidylinositol-4-phosphate in the plasma membrane

**DOI:** 10.1080/19768354.2019.1599424

**Published:** 2019-04-11

**Authors:** Yong-Woo Jun, Jin-A Lee, Deok-Jin Jang

**Affiliations:** a Department of Ecological Science, College of Ecology and Environment, Kyungpook National University, Sangju-si, Republic of Korea; b Department of Biological Science and Biotechnology, College of Life Science and Nano Technology, Hannam University, Daejeon, Republic of Korea

**Keywords:** Phosphoinositide, PI4P, PI(4,5)P_2_, PH domain, plasma membrane

## Abstract

Phosphatidylinositol-4-phosphate (PI4P) plays a crucial role in cellular functions, including protein trafficking, and is mainly located in the cytoplasmic surface of intracellular membranes, which include the trans-Golgi network (TGN) and the plasma membrane. However, many PI4P-binding domains of membrane-associated proteins are localized only to the TGN because of the requirement of a second binding protein such as ADP-ribosylation factor 1 (ARF1) in order to be stably localized to the specific membrane. In this study, we developed new probes that were capable of detecting PI4P at the plasma membrane using the known TGN-targeting PI4P-binding domains. The PI4P-specific binding pleckstrin homology (PH) domain of various proteins including CERT, OSBP, OSH1, and FAPP1 was combined with the N-terminal moderately hydrophobic domain of the short-form of *Aplysia* phosphodiesterase 4 (S(N30)), which aids in plasma membrane association but cannot alone facilitate this association. As a result, we found that the addition of S(N30) to the N-terminus of the GFP-fused PH domain of OSBP (S(N30)-GFP-OSBP-PH), OSH1 (S(N30)-GFP-OSH1-PH), or FAPP1 (S(N30)-GFP-FAPP1-PH) could induce plasma membrane localization, as well as retain TGN localization. The plasma membrane localization of S(N30)-GFP-FAPP1-PH is mediated by PI4P binding only, whereas those of S(N30)-GFP-OSBP-PH and S(N30)-GFP-OSH1-PH are mediated by either PI4P or PI(4,5)P_2_ binding. Taken together, we developed new probes that detect PI4P at the plasma membrane using a combination of a moderately hydrophobic domain with the known TGN-targeting PI4P-specific binding PH domain.

## Introduction

Phosphoinositides, which are derivatives of phosphatidylinositol (PI), are minor lipid components of eukaryotic membranes, but have important roles in many cellular functions, including serving as lipid markers of intracellular membrane organelles (Di Paolo and De Camilli 2006; Lemmon 2008). Phosphoinositides are not randomly distributed within intracellular membranes, but rather, each type has a unique cellular localization (Di Paolo and De Camilli 2006; Jang et al. 2009). For example, phosphatidylinositol 3-phosphate (PI3P) is localized to the cytoplasmic surface of early endosomes, while phosphatidylinositol 4-phosphate (PI4P) is mainly enriched in the trans-Golgi network (TGN) of cells (D'Angelo et al. 2008).

PI4P is mostly known to serve as a precursor of phosphatidylinositol 4,5-bisphosphate (PI(4,5)P_2_). However, PI4P plays many roles in the plasma membrane. For example, PI4P is involved in the plasma membrane targeting of many plasma membrane proteins through direct binding or electrostatic interactions (Hammond et al. 2012; Simon et al. 2016). Depletion of both PI4P and PI(4,5)P_2_ simultaneously could change the localization of many plasma membrane-targeted proteins, such as the short forms of *Aplysia* phosphodiesterase 4 (PDE4) and K-Ras in animal cells (Jang et al. 2010; Hammond et al. 2012; Kim et al. 2014). In plant cells, PI4P is highly enriched in the plasma membrane and plays a key role in the formation of membrane surface charges that lead to the recruitment of a variety of membrane proteins (Simon et al. 2016). PI4P is indirectly involved in the enrichment of phosphatidylserine (PS) in the plasma membrane through oxysterol-binding protein (OSBP)-related protein 5 (ORP5) and ORP8 (Chung et al. 2015; Sohn and Korzeniowski 2018).

**Figure 1.**
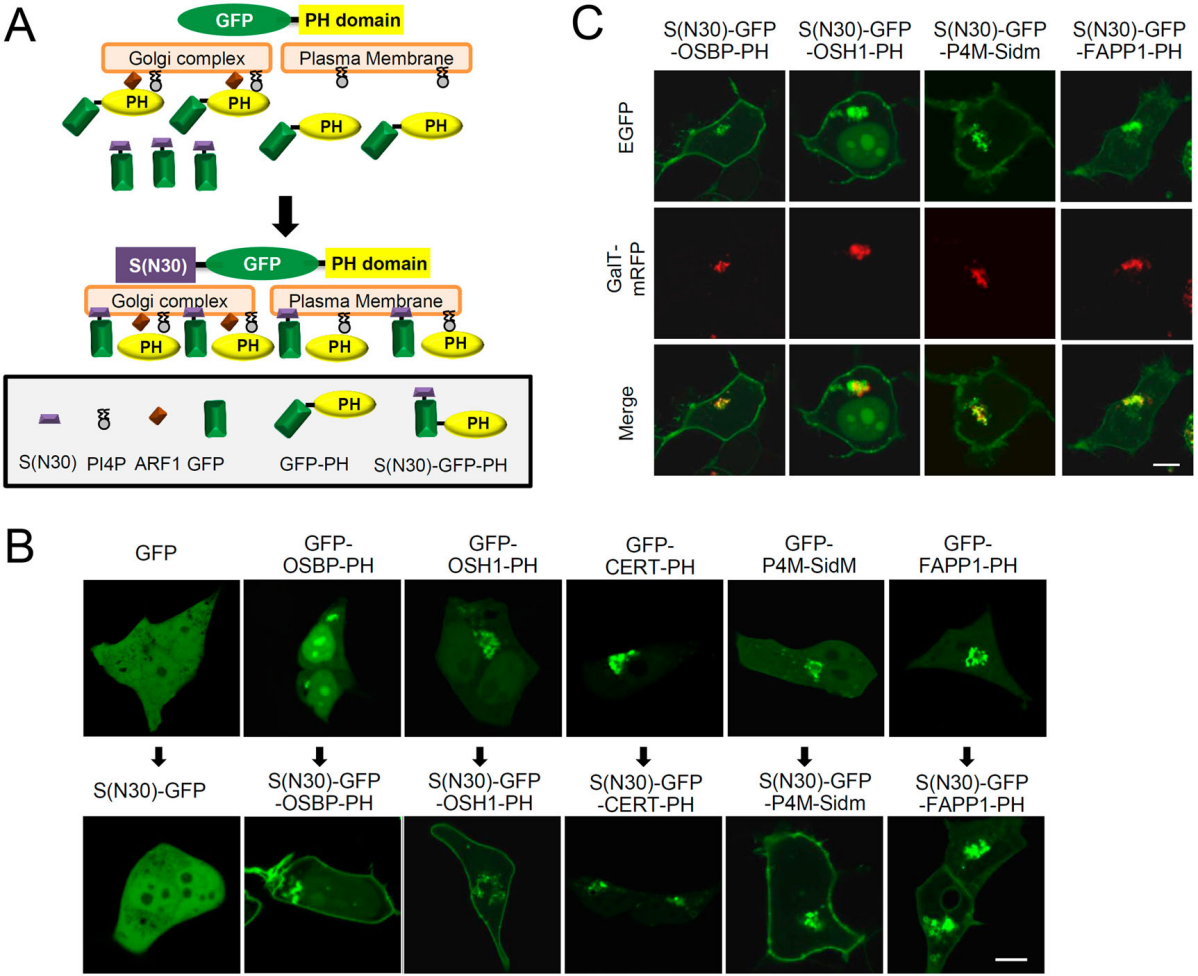
Plasma membrane localization of S(N30)-GFP-X proteins. (A) Schematic model of the development of new sensors detecting PI4P at the plasma membrane. (B) Confocal images showing cellular localization of various GFP-X (upper) and S(N30)-GFP-X constructs (lower) in HEK293 T cells. (C) Trans-Golgi network (TGN) localization of various S(N30)-GFP-X. GalT-mRFP was used as a TGN marker in HEK293 T cells. X: OSBP-PH, OSH1-PH, CERT-PH, FAPP1-PH, and P4M-SidM.; Y: OSBP-PH, OSH1-PH, FAPP1-PH, and P4M-SidM. Scale bar, 20 μm.

Until recently, although PI4P is located at the cytoplasmic surface of the TGN, plasma membrane, and endosome, most PI4P-binding probes were only localized to the TGN. For example, the PH domains of OSH1, four-phosphate-adaptor protein (FAPP), and oxysterol-binding protein (OSBP) are PI4P-binding proteins, but they require other binding proteins, including ADP ribosylation factor 1 (ARF1), to be localized to the TGN (Hanada et al. 2003; Godi et al. 2004; Roy and Levine 2004). Recently, we found that an N-terminal hydrophobic domain itself was not sufficient for specific membrane organelle targeting, but additional domains induced the stable plasma membrane or autopahgosome association of the ApPDE4 short-form (Kim et al. 2014; Lee et al. 2017). Therefore, we hypothesized that if the N-terminal hydrophobic domain of the ApPDE4 short-form, which itself was localized in the cytosol but helps the plasma membrane localization, was combined with the PI4P-binding PH domains, these novel PI4P-specific probes could detect PI4P at the plasma membrane and endosome (Figure 1A).

In this study, we found that if an N-terminal hydrophobic domain of ApPDE4 short-form is linked to the GFP-fused PH domain of OSBP, OSH1, and FAPP1, these proteins were localized to the plasma membrane as well as the TGN. The plasma membrane localization of modified OSBP and OSH1 is mediated by PI4P as well as PI(4,5)P_2_, whereas a modified PH domain FAPP1 was localized to the plasma membrane and endosome mainly via PI4P binding. Thus, we could develop new probes detecting PI4P at the plasma membrane using a combination of the moderately hydrophobic domain with the known PI4P-specific binding PH domain.

## Materials and methods

### Constructs and plasmids

All primers are described in Table 1. The region encoding the N-terminal moderately hydrophobic domain of the *Aplysia* PDE4 short-form (S(N30)) was amplified by polymerase chain reaction (PCR) with apPDE (short)-D3-S/Short (N30)-Xba1-A primer set and inserted between the HindIII and XbaI sites of the pcDNA3.1(+)-GFP vector. GFP-OSBP-PH, OSH1-PH, and FAPP1-PH were kindly provided by Tamas Balla (National Institutes of Health). GFP-P4M-SidM was obtained from Addgene (Cambridge, MA, USA). To generate pcDNA3.1(+)-S(N30)-GFP-OSBP-PH and -OSH1-PH, GFP-OSBP-PH or GFP-OSH1-PH was amplified by PCR with GFP-XbaI-S/pEGFP-A primer set. To generate pcDNA3.1(+)-S(N30)-GFP-CERT-PH, -P4M-SidM, or -FAPP1-PH, GFP-CERT-PH, -P4M-SidM, or -FAPP1-PH was amplified by PCR with GFP-XbaI-S/hCERT-ApaI-A, GFP-XbaI-S/P4M-SidM-Apa I-A, or GFP-XbaI-S/FAPP1-ApaI-A pEGFP-A primer set. Each PCR product was separately inserted between the XbaI-ApaI sites of the pcDNA3.1(+)-S(N30)-GFP vector. The pseudojanin (PJ) assay system was described previously (Kim et al. 2014; Jun et al. 2015).

**Table 1.** Primer sequences used for PCR. S, sense primer; A, anti-sense primer.

Name	Primer Sequence (5′–3′)
apPDE (short)-D3-S	CGAAGCTTGCCACCACCATGCAGAAGCTGAATTTC
Short (N30)-Xba1-A	GCTCTAGAATCAGTTGAACTCTTCCT
GFP-XbaI-S	GCTCTAGAATGGTGAGCAAGGGCGAG
OSBP, OSH1 (pEGFP-A)	GGGAGGTGTGGGAGGTTTT
hCERT-ApaI-A	CGTAGGGCCCTTATGCTCCAGACACCAG
P4M-SidM-Apa I-A	CGTAGGGCCCTTATTTTATCTTAATGGT
FAPP1-ApaI-A	CGTAGGGCCCTTATTTAGTCCTTGTATC

### HEK293 T cell culture and confocal microscopy

HEK293 T cells were grown in Dulbecco’s modified Eagle’s medium (DMEM) supplemented with 10% (v/v) fetal bovine serum and penicillin/streptomycin in a humidified atmosphere of 5% (v/v) CO_2_ at 37°C. Cells were seeded in a sticky-Slide 8-well system (Catalog #: 80828, Ibidi, Martinsried, Germany), in order to reach 40%–60% confluency on the day of imaging. In the period 24–26 h before imaging, the cells were transfected with DNA constructs using calcium phosphate or Lipofectamine 2000 (Life Technologies, Carlsbad, CA, USA). The relative amount of each construct was empirically determined based on the relative expression of each construct combination. Cells were then analyzed using an inverted Zeiss LSM-700 confocal scanning laser microscope operated by ZEN software (Carl Zeiss). Most images were derived from live cells.

### Drug treatment

ATP depletion was performed by incubating cells with 200 nM antimycin A (Sigma-Aldrich, catalog number: A8674) in a calcium- and glucose-free medium (PBS) (Sigma-Aldrich, catalog number: P5368) for at least 40 min to delete cellular PI derivatives such as PI4P, PI(4,5)P_2_, and PI(3,4,5)P_3_. Cells expressing FKBP-related constructs, mRFP-FKBP-PJ, INPP5E, PJ-Sac, PJ-Dead, FAPP-PH, and OSH1-PH, were treated with 1 μM rapamycin (Sigma-Aldrich, catalog number: R8781) in culture medium for 1–3 min.

## Results and discussion

### Generation of modified PI4P binding probes localized to the plasma membrane

We first examined the cellular localization of the GFP-fused PH domain of OSBP (GFP-OSBP-PH), OSH1 (GFP-OSH1-PH), CERT (GFP-CERT-PH), or FAPP1 (GFP-FAPP1-PH), which were known as TGN-targeting PI4P binding domains (Hanada et al. 2003; Godi et al. 2004; Roy and Levine 2004) (Figure 1B, upper). We also used the GFP-fused P4M domain of SidM (GFP-P4M-SidM), which was recently reported as a more specific PI4P binding protein and could detect PI4P at the plasma membrane as well as the TGN (Hammond et al. 2014). As previously reported, GFP-P4M-SidM was mainly localized to the TGN and weakly to the plasma membrane (Figure 1B, upper). Thus, except for GFP-P4M-SidM, all other probes were not localized to the plasma membrane.

Next, as hypothesized, we added the N-terminal hydrophobic domain (N-terminal 30 amino acids) of the short form of ApPDE4 onto the N-terminus of GFP in each PI4P binding probe, generating S(N30)-GFP-OSBP-PH, S(N30)-GFP-OSH1-PH, S(N30)-GFP-CERT-PH, S(N30)-GFP-FAPP1, and S(N30)-GFP-P4M-SidM. Each construct was expressed in HEK293 T cells alone (Figure 1B, lower) or co-expressed with GalT-mRFP, a TGN marker (Figure 1C). Interestingly, although S(N30)-GFP alone was localized to the cytosol, S(N30)-GFP-OSBP-PH, S(N30)-GFP-OSH1-PH, and S(N30)-GFP-FAPP1-PH were clearly localized to the plasma membrane as well as the TGN (Figure 1B, lower and C). In the case of S(N30)-GFP-P4M-SidM, it was localized to the plasma membrane more than GFP-P4M-SidM. Meanwhile, S(N30)-GFP-CERT-PH was localized to only the TGN, as was GFP-CERT-PH. Taken together, our results showed that the addition of the N-terminal hydrophobic domain of the ApPDE4 short-form into the PI4P-binding domains could induce plasma membrane targeting as well as retain TGN targeting.

### Effect of an acute depletion of phosphoinositides on cellular localization of modified PI4P-binding domains

**Figure 2.**
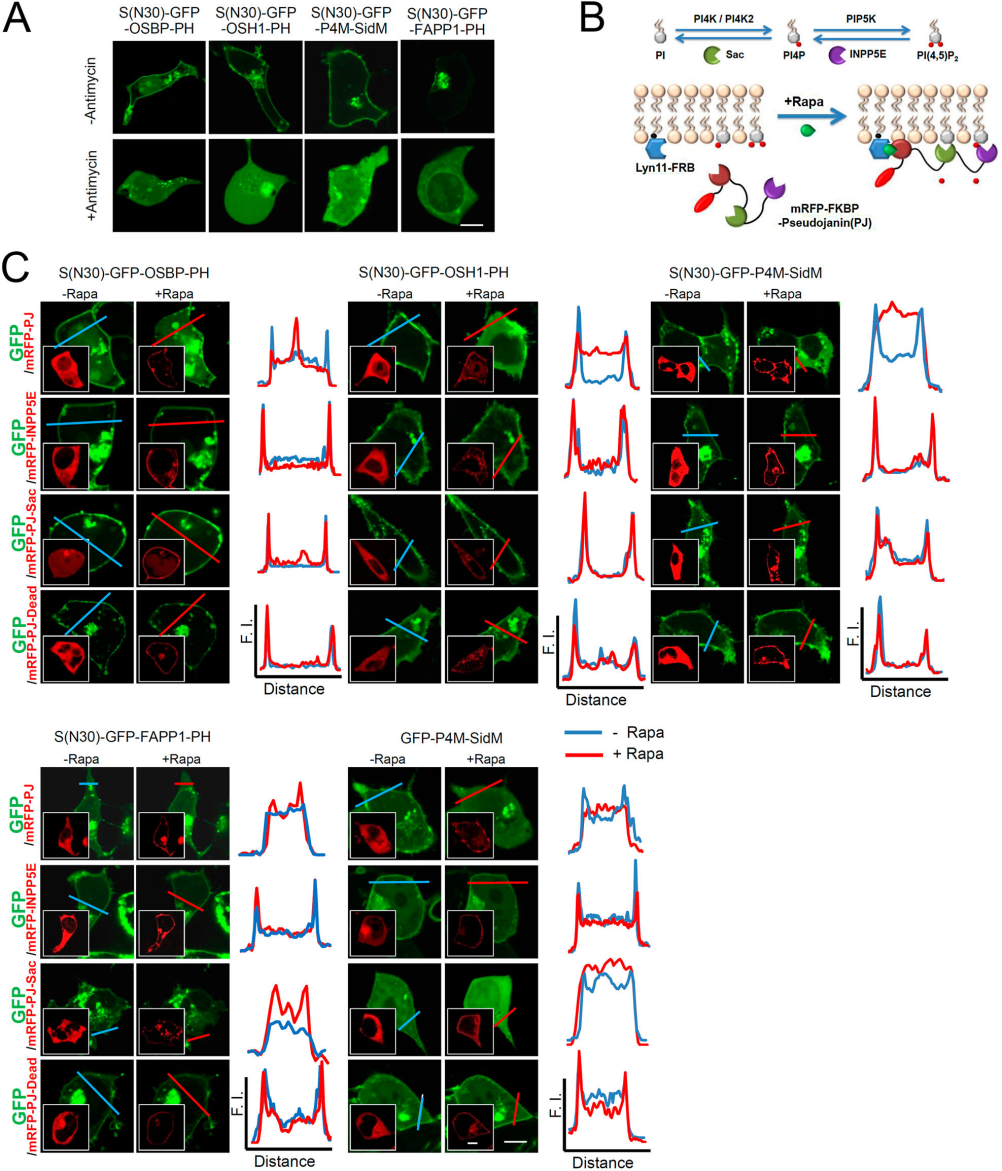
Dissection of the molecular mechanism of the plasma membrane targeting of S(N30)-GFP-X proteins. (A) Effects of phosphoinositide depletion by antimycin A treatment. Various GFP-fused proteins are expressed in HEK293 T cells. (B) A schematic diagram of the experimental models of the Lyn11-FRB/PJ system. Yeast Sac1 dephosphorylates PI4P and INPP5E and converts PI(4,5)P_2_ to PI4P (upper). In the absence of rapamycin (Rapa), lyn11-FRB and mRFP-FKBP-PJ are localized to the plasma membrane and cytosol, respectively (lower). In the presence of Rapa, Rapa-FRB can be associated through FKBP, leading to plasma membrane targeting of mRFP-FKBP-PJ, depleting PIs. (C) Confocal images showing cellular localization of S(N30)-GFP-X proteins in the absence or presence of rapamycin in HEK293 T cells. The colored lines in the confocal fluorescence images indicate the paths along which the fluorescence intensities (F.I.) were plotted to the right. S(N30)-GFP-X, or GFP-P4M-SidM was co-transfected with mRFP-PJ, mRFP-PJ-Sac, mRFP-INPP5E, and mRFP-PJ-Dead. Plasma membrane localization of the short-form is switched to the cytoplasm by mRFP-PJ, but not by mRFP-PJ-Sac, mRFP-INPP5E, or mRFP-PJ-DEAD recruitment. GFP-P4M-SidM was used as a control. X: OSBP-PH, OSH1-PH, P4M-SidM, and FAPP1-PH. Scale bar, 20 μm.

Next, to determine whether phosphoinositides are involved in the plasma membrane localization of these probes, antimycin A, an ATP synthesis inhibitor, was used to deplete cellular lipid derivatives generated by various lipid kinases, including various phosphoinositides such as PI4P, PI(4,5)P_2_, and PI(3,4,5)P_3_ within cells (Kim et al. 2014). As shown in Figure 2A, in the presence of antimycin A, the plasma membrane localization of S(N30)-GFP-OSBP-PH, S(N30)-GFP-OSH1-PH, S(N30)-GFP-FAPP1-PH, and S(N30)-GFP-P4M-SidM was disrupted (Figure 2A). In the case of S(N30)-GFP-FAPP1-PH, there was weak localization to the nuclear membrane in the presence of antimycin A (Figure 2A). Taken together, these results indicate the possibility that the plasma membrane localization of GFP-fusion proteins is probably mediated by phosphoinositide binding.

Next, to examine which phosphoinositides are involved in the plasma membrane targeting of the modified probes, we used the pseudojanin (PJ) system for transient membrane phosphoinositide depletion in living cells (Suh et al. 2006; Kim et al. 2014). Each modified probe was co-transfected with Lyn11-FRB, the rapamycin-binding domain of mTOR and mRFP-FKBP, the FK506 binding protein 12-PJ, which can be recruited to the plasma membrane in an inducible manner in response to rapamycin treatment. PJ contains active yeast Sac1, which dephosphorylates PI4P, and active polyphosphate 5-phosphatase E (INPP5E), which converts PI(4,5)P_2_ to PI4P. Together they can deplete PI4P and PI(4,5)P_2_ (Figure 2B). Others were PJ-Sac, which contains active Sac1 and inactive INPP5E, INPP5E, containing inactive Sac1 and active INPP5E, and PJ-Dead, containing inactive Sac1 and inactive INPP5E (Hammond et al. 2012). Using these methods, we could discriminate which phosphoinositides are key players in targeting the new probes to the plasma membrane. As a result, we found that the localization of S(N30)-GFP-OSBP-PH, S(N30)-GFP-OSH1-PH, and S(N30)-GFP-P4M-SidM was shifted from the plasma membrane to the cytosol by rapamycin treatment in cells expressing mRFP-FKBP-PJ (Figure 2C). Meanwhile, the localization of S(N30)-GFP-OSBP-PH, S(N30)-GFP-OSH1-PH, and S(N30)-GFP-P4M-SidM was not shifted from the plasma membrane to the cytosol by rapamycin treatment in cells expressing mRFP-FKBP-Inpp5E, mRFP-FKBP-PJ-Sac, or mRFP-FKBP-PJ-Dead (Figure 2C). On the other hand, the localization of S(N30)-GFP-FAPP1-PH and GFP-P4M-SidM was shifted from the plasma membrane to the cytosol by rapamycin treatment in cells expressing mRFP-FKBP-PJ and mRFP-FKBP-PJ-sac, but not in cells expressing mRFP-FKBP-Inpp5E and mRFP-FKBP-PJ-Dead. These results indicate that S(N30)-GFP-OSBP-PH, S(N30)-GFP-OSH1-PH, and S(N30)-GFP-P4M-SidM were localized to the plasma membrane via either PI4P or PI(4,5)P_2_ binding, while GFP-P4M-SidM and S(N30)-GFP-FAPP1-PH were localized to the plasma membrane mainly through PI4P binding.

### Generation of inducible PI4P binding probes localized to the plasma membrane

**Figure 3.**
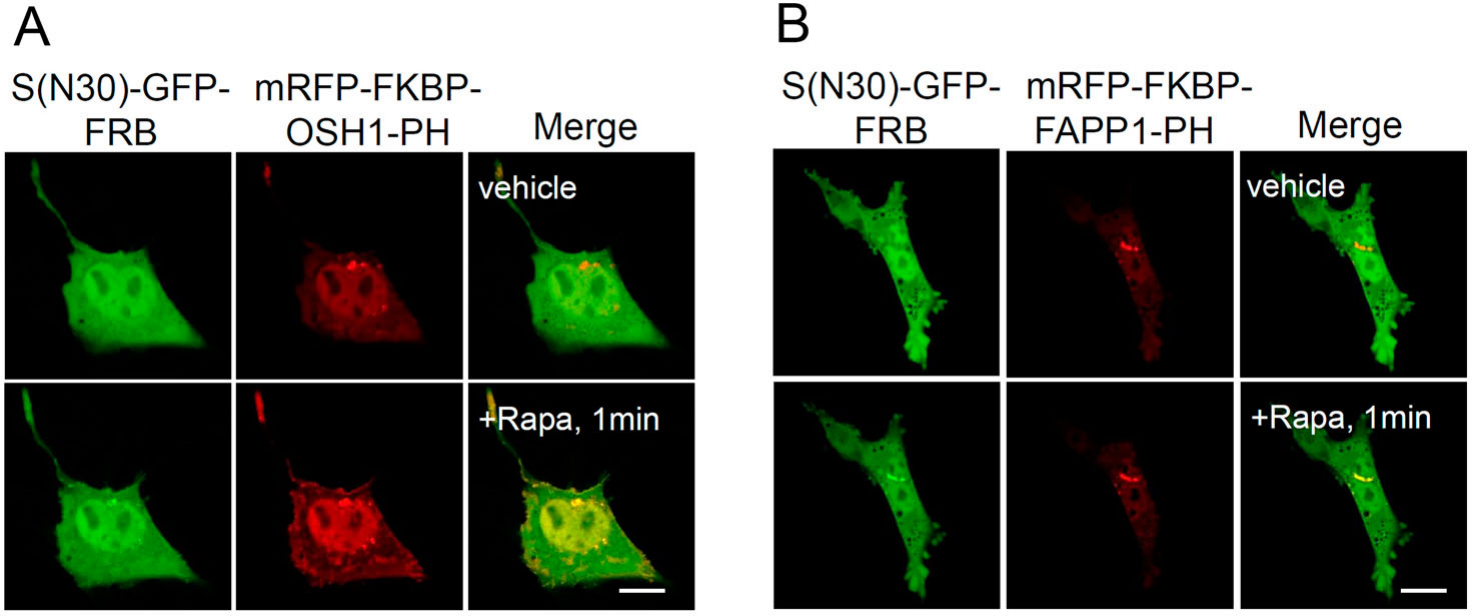
Generation of an inducible PI4P binding probe at the plasma membrane. Confocal images showing cellular localization of cells expressing S(N30)-GFP-FRB and mRFP-FKBP-OSH1-PH (A) or expressing S(N30)-GFP-FRB and mRFP-FKBP-FAPP1-PH (B) in the absence (upper) or presence of rapamycin (lower) in HEK293 T cells. Scale bar, 20 μm.

To generate inducible probes detecting PI4P at the plasma membrane, we applied the FRB/rapamycin/FKBP system as shown in Figure 2B. To this end, we generated S(N30)-GFP-FRB, mRFP-FKBP-OSH1-PH, and mRFP-FKBP-FAPP1-PH. S(N30)-GFP-FRB was then co-expressed with mRFP-FKBP-OSH1-PH or mRFP-FKBP-FAPP1-PH in HEK293 T cells. As shown in Figure 3, in the absence of rapamycin, S(N30)-GFP-FRB was expressed in the cytosol and mRFP-FKBP-OSH1-PH or mRFP-FKBP-FAPP1-PH was localized to the cytosol and TGN. However, in the presence of rapamycin, S(N30)-GFP-FRB and mRFP-FKBP-OSH1-PH were weakly localized to the plasma membrane well as the TGN in cells expressing S(N30)-GFP-FRB and mRFP-FKBP-OSH1-PH (Figure 3A). On the other hand, S(N30)-GFP-FRB was localized to only the Golgi complex but not to the plasma membrane in cells expressing S(N30)-GFP-FRB and mRFP-FKBP-FAPP-PH in the presence of rapamycin (Figure 3B). However, compared to S(N30)-GFP-OSH1-GFP, the plasma membrane localization of S(N30)-GFP-FRB and mRFP-FKBP-OSH1-PH in the presence of rapamycin was very weak (Figures 2 and 3).

Overall, this study demonstrates that by adding the N-terminal hydrophobic domain of the short form of ApPDE4 to the known TGN-targeted PI4P binding domain, a probe capable of PI4P detection in the plasma membrane was generated. These constructs will be useful for future PI4P research.
